# Exposure of Human CD8^+^ T Cells to Type-2 Cytokines Impairs Division and Differentiation and Induces Limited Polarization

**DOI:** 10.3389/fimmu.2018.01141

**Published:** 2018-05-28

**Authors:** Annette Fox, Kim L. Harland, Katherine Kedzierska, Anne Kelso

**Affiliations:** Department of Microbiology and Immunology, Peter Doherty Institute for Infection and Immunity, University of Melbourne, Melbourne, VIC, Australia

**Keywords:** CD8^+^ T cell, human, cytokine, IFN-γ, IL-4, differentiation and reprogramming, plasticity

## Abstract

Effector CD8^+^ T cells generally produce type-1 cytokines and mediators of the perforin/granzyme cytolytic pathway, yet type-2-polarized CD8^+^ cells (Tc2) are detected in type-2 (T2) cytokine-driven diseases such as asthma. It is unclear whether T2 cytokine exposure during activation is sufficient to polarize human CD8^+^ T cells. To address this question, a protocol was developed for high-efficiency activation of human CD8^+^ T cells in which purified single cells or populations were stimulated with plate-bound anti-CD3 and anti-CD11a mAb for up to 8 days in T2 polarizing or neutral conditions, before functional analysis. Activation of CD8^+^ naïve T cells (T_N_) in T2 compared with neutral conditions decreased the size of single-cell clones, although early division kinetics were equivalent, indicating an effect on overall division number. Activation of T_N_ in T2 conditions followed by brief anti-CD3 mAb restimulation favored expression of T2 cytokines, GATA3 and *Eomes*, and lowered expression of type-1 cytokines, *Prf1*, Gzmb, T-BET, and *Prdm1*. However, IL-4 was only weakly expressed, and PMA and ionomycin restimulation favored IFN-γ over IL-4 expression. Activation of T_N_ in T2 compared with neutral conditions prevented downregulation of costimulatory (CD27, CD28) and lymph-node homing receptors (CCR7) and CD95 acquisition, which typically occur during differentiation into effector phenotypes. CD3 was rapidly and substantially induced after activation in neutral, but not T2 conditions, potentially contributing to greater division and differentiation in neutral conditions. CD8^+^ central memory T cells (T_CM_) were less able to enter division upon reactivation in T2 compared with neutral conditions, and were more refractory to modulating IFN-γ and IL-4 production than CD8^+^ T_N._ In summary, while activation of T_N_ in T2 conditions can generate T2 cytokine-biased cells, IL-4 expression is weak, T2 bias is lost upon strong restimulation, differentiation, and division are arrested, and reactivation of T_CM_ is reduced in T2 conditions. Taken together, this suggests that exposure to T2 cytokines during activation may not be sufficient to generate and retain human Tc2 cells.

## Introduction

Effector CD4^+^ T cells are remarkably heterogeneous (1), consistent with the capacity of naïve T cells (T_N_) to tailor differentiation according to which cytokines are present during activation (2, 3). Most notably IFN-γ and IL-4 promote differentiation into T helper 1 (Th1) and 2 (Th2) subsets, respectively. Effects of IFN-γ and IL-4 are orchestrated by induction of distinct transcription factors: T-BET and GATA3. These transcription factors participate in chromatin remodeling whereby histone modifications and DNA methylation alter genome accessibility to transcription. T-BET is induced by IFN-γ and increases Th1 locus accessibility while decreasing Th2 locus accessibility, whereas GATA3 is induced by IL-4 and has opposing effects on Th1 and Th2 loci (1, 2). The cytokine-induced epigenetic changes that occur during T-cell priming generate memory T cells that are programmed to produce type-1 or type-2 cytokines upon recall. However, CD4^+^ T central memory cells (T_CM_) retain some functional flexibility and capacity to adapt to a different recall environment (3, 4).

Studies of mouse CD8^+^ T_N_ indicate a predisposition to differentiate into IFN-γ producing cells (5). Nevertheless, if mouse CD8^+^ T_N_ are primed in a type-2 (T2) cytokine environment, cytolytic function and IFN-γ expression are suppressed, while IL-5, IL-13, and, to a lesser extent, IL-4 are induced (6–10). Importantly, single cell and paired daughter cell analyses indicate that a large proportion of murine CD8^+^ T_N_ cells have the potential to produce T2 cytokines, and some retain this potential after extensive expansion in neutral conditions (8). As may be expected, T2-primed CD8^+^ T cells have reduced capacity to clear influenza virus upon transfer to infected recipient mice compared with neutral primed CD8^+^ T cells (11). Virus-specific CD8^+^ T cells with T2 functions (IL-5 and IL-13 production) are also induced upon *in vivo* priming in the presence of IL-4 (12, 13), but the fate of these cells is unclear. Some studies indicate that priming in T2 conditions generates memory cells that retain T2 function when recalled (14). In contrast, others indicate that recalled T2-primed CD8^+^ T cells revert to type-1 cytokine production and anti-tumor function unless repeatedly activated in T2 conditions (15). It is important to understand whether effects of T2 conditions on CD8^+^ T cells are maintained long term and whether they are replicated in human CD8^+^ T cells as this may imply that early life, T2 cytokine-associated events, such as asthma, could have lasting effects on the anti-viral capacity of CD8^+^ T cells.

CD8^+^ T cells that produce IL-4, but little or no IFN-γ, can be detected in patients with Th2-associated diseases (16–20). T2 cytokine-biased CD8^+^ T cells (Tc2) can be cloned from the blood of these patients, but not from healthy human donors (16–18). Studies conducted in the 1990s found that human cord blood CD8^+^ T cells produced little IL-4, measured by ELISA, when activated in the presence of IL-4 (21). This raises the question as to how Tc2 cells arise: do human naïve CD8^+^ T cells readily differentiate into T2 cytokine producing cells if T2 cytokines are present during activation, or does the differentiation of Tc2 cells require continued exposure to T2 cytokines? This study assesses the effect of exposure to a T2 cytokine environment during human CD8^+^ T-cell activation. Single-cell cloning facilitates measurement of plasticity within a cell population, and cloning in the absence of feeder cells allows the effects of different stimuli to be assessed without interference from signals provided by the feeder cells. Others have developed a feeder cell-free system with around 40% efficiency for cloning single human CD8^+^ T cells (22). We developed a more efficient system that was used, in combination with bulk culture, to demonstrate that purified human CD8^+^ naïve T cells (T_N_) could be T2 polarized by activation in T2 conditions, but at a cost to division and differentiation. In addition, we have shown that T2 conditions prevent reactivation of CD8^+^ central memory T cells (T_CM_), and that T_CM_ are more refractory than T_N_ to polarization.

## Materials and Methods

### Participants and Samples

Venous blood samples were collected from healthy volunteers who had provided written informed consent as part of a project approved by the University of Melbourne Health Sciences Human Ethics Sub-Committee (#1443389). PBMCs were isolated from heparinized blood *via* Lymphoprep (Stemcell Technologies) gradient.

### Fluorescence-Activated Cell Sorting and Analysis

PBMCs were stained with Live/Dead Fixable Dead Cell Stain Kit (Invitrogen, Molecular Probes) followed by fluorochrome-conjugated mAbs to: CD3ε (HIT3a), CD8α (RPA-T8), CD4 (RPA-T4), CD28 (CD28.2), CD95 (DX2) from BD; CD45RA (HI100), CD27 (O323), CD197 (G043H7), IFN-γ (4S.B3), IL-4 (8D4-8), TNF-α (Mab11), and isotype controls from Biolegend; CD3ε (UCHT1), CD62L (DREG-56), GATA3 (TWAJ), T-BET (4B10), and isotype control with Foxp3 Staining Buffer Set from eBioscience; Granzyme B (GB12) and mAb isotype control from Invitrogen.

CD3^+^ CD8^+^ T cells were sorted into T_N_ (CD45RA^hi^ CD27^hi^ CD62L^hi^ CD95^lo^) and T_CM_ (CD45RA^lo^ CD27^+^) subsets using an FACS Aria III (Figure S1 in Supplementary Material). Further analysis demonstrated that cells in the T_N_ gate were CCR7^hi^ while those in the T_CM_ gate were CCR7^int^ CD95^lo^, and heterogeneous for CD62L (Figure S1 in Supplementary Material). Sorted cell purities were more than 95%.

### *In Vitro* Activation of Purified CD8^+^ T-Cell Subsets

T_N_ (5,000–10,000) or T_CM_ (20,000) were added to 24-well plates coated with anti-CD3 mAb (clone OKT3, purchased from the Walter and Eliza Hall Institute, Parkville, Australia) at 1 µg/mL and anti-CD11a mAb (clone MEM-83, generous gift from Vaclav Horejsi, Institute of Molecular Genetics, Prague, Czech Republic) at 19 µg/mL unless otherwise indicated. Cells were incubated in culture medium (RPMI supplemented with 9% FBS, 2-mM L-glutamine, 1-mM MEM sodium pyruvate, 100-U/mL penicillin/streptomycin, 100-µM MEM non-essential amino acids, 5-mM HEPES buffer, and 55-µM 2-mercaptoethanol). rHuman IL-2 (Roche 11 147 528 001) was added at 25 U/mL for neutral and T2 conditions. T2 cultures also contained 10-ng/mL rHuman IL-4 (Shenandoah Cat. #100-09) and 1-µg/mL antihuman IFN-γ mAb (Biolegend Cat. #502404). Antihuman IL-12p70 mAb clone QS12p70 (U-Cy Tech biosciences Cat. #CT276) was added to T2 media at 1 µg/mL in one experiment. Cells were recovered from stimulation plates after 5 days and assessed directly or rested in fresh plates for 3 days in culture medium containing IL-2.

### Division Analysis

Sorted cells were resuspended in 1 mL of PBS containing 1 µL of Violet Proliferation Dye 450 (BD Biosciences, Cat. #562158), incubated in a 37°C waterbath for 30 min then washed and resuspended at 50,000 per well for analysis within 85 h, and at 40,000 per well for analysis at 90 h.

### Single-Cell Cloning of CD8^+^ T Cells

Protocols for single-cell cloning of mouse CD8^+^ T cells in the absence of feeder cells were adapted for cloning human CD8^+^ T cells (23). Purified cells were sorted individually into wells of 96-well round-bottom plates coated with OKT3 and MEM-83 mAbs, as above, and containing 160 µL of neutral or T2 culture medium. One plate (96 cells) was prepared per subset, condition, and donor unless otherwise indicated. Cloning efficiency and clone size were determined by microscopic scoring of proliferation on day 6.

### Restimulation for Functional Analysis

Rested cells were restimulated for 5 h in culture medium with either 10-ng/mL phorbol 12-myristate 13-acetate (PMA, Sigma Cat. #P1585) and 500-ng/mL ionomycin (Sigma Cat. #I0634), 1-µg/mL plate-bound anti-CD3 mAb, or 1-µg/mL plate-bound anti-CD3 with 10-µg/mL anti-CD11a mAb. Cells were split, to be used for either RNA extraction then RT-PCR, or intracellular staining, in which case Golgi-stop (0.67 µl/mL; BD Biosciences) was added. To detect intracellular cytokines and transcription factors, cells were first stained for surface markers then fixed and permeabilized using BD Cytofix/Cytoperm kit (BD Biosciences) and Foxp3/Transcription Factor Staining Buffer Set (eBioscience), respectively. Cells were then incubated with cytokine or transcription factor specific mAbs diluted in Perm/Wash Buffer (BD Biosciences). Post-acquisition data analyses were performed using FlowJo software (Tree Star).

### Real-Time RT-PCR

RNA was extracted from fresh cell pellets using RNeasy Mini Kit (QIAGEN) and reverse transcribed using SuperScript^®^ VILO cDNA Synthesis Kit (Invitrogen). cDNA was diluted to obtain similar levels of ribosomal protein S18 (RPS18) reference gene, then 1 µL was added to 20-µL PCR reactions containing 200 nM of each primer (Table 1), Taq DNA Polymerase and buffer (QIAGEN), and SYTO 9 green fluorescent nucleic acid stain (Molecular Probes, Cat. #S34854). Real-time PCR was performed on a CFX96 (Bio-Rad) for 5 min at 95°C followed by 45 cycles of 95°C for 30 s, 61°C for 1 min, 72°C for 1 min, and melting at 50–95°C. RPS18 reference gene was run together with each target to normalize target gene levels relative to mRNA levels.

**Table 1:** Primer sequences.

Gene		Primer sequence
*CD27*	Forward	TGTCGGCACTGTAACTCTGGTCT
	Reverse	CCTGCACTGCCAGCCAT
*RPS18*	Forward	ACTCACTGAGGATGAGGTGGAA
	Reverse	ATTGGCTAGGACCTGGCTGTAT
*Cd8a*	Forward	CAGCGGTTCTCGGGCAA
	Reverse	GTGGTGGGCTTCGCTGG
*Ifng*	Forward	GCTGACTAATTATTCGGTAACTGACTTG
	Reverse	GCGACAGTTCAGCCATCACTT
*Il4*	Forward	GGCGGGCTTGAATTCCT
	Reverse	TCCAAGAAGTTTTCCAACGTACTCT
*RUNX3*	Forward	CGAGGGAAGAGTTTCACCCTGAC
	Reverse	AGGTGCCTTGGATTGGGGTCT
*Eomes*	Forward	CAGGCGCATGTTTCCTTTCTTG
	Reverse	TGAACATACATTTTGTTGCCCTGC
*GATA3*	Forward	CGGTCCAGCACAGGCA
	Reverse	GCAGACAGCCTTCGCTTG
*Gzmb*	Forward	AAGGCCCAGGTGAAGCCAG
	Reverse	GGCCTCCAGAGTCCCCCTTA
*Prf1*	Forward	GGTGGAGTGCCGCTTCTACAGTT
	Reverse	GGATGAAGTGGGTGCCGTAGTTG
*TBX21*	Forward	CGAGATTACTCAGCTGAAAATTGAT
	Reverse	TGTCAACAGATGTGTACATGGACT
*Prdm1*	Forward	CAATGATGAATCTCACACAAACACAGA
	Reverse	GAGGGTAGAAGGGCATTTCGG
*IL5*	Forward	GAACTCTGCTGATAGCCAATGAGAC
	Reverse	ATTTCTTCAGTGCACAGTTGGTGAT

### Analysis

SPSS, Prism, and Excel were used to present data graphically and to perform statistical analysis comparing effects of neutral (N) versus T2 conditions on responses within each subset, with and without restimulation. Shapiro–Wilks and Kolmogorov–Smirnov tests were used to assess data normality for each parameter, subset, and condition compared. Where necessary and possible, data were normalized by log-transformation, then assessed using ANOVA with Bonferroni’s correction for multiple pairwise comparisons. Pairwise comparisons that reached *p* < 0.1 were finally assessed using paired *t*-test. Data that failed normality testing following log-transformation were assessed using non-parametric Kruskal–Wallis Test with Dunn’s correction for multiple comparisons. Pairwise comparisons that reached *p* < 0.1 were finally assessed using Wilcoxon Rank-Sum Test for paired data.

### Optimizing Conditions for *In Vitro* Activation and Single-Cell Cloning

**Figure 1:**
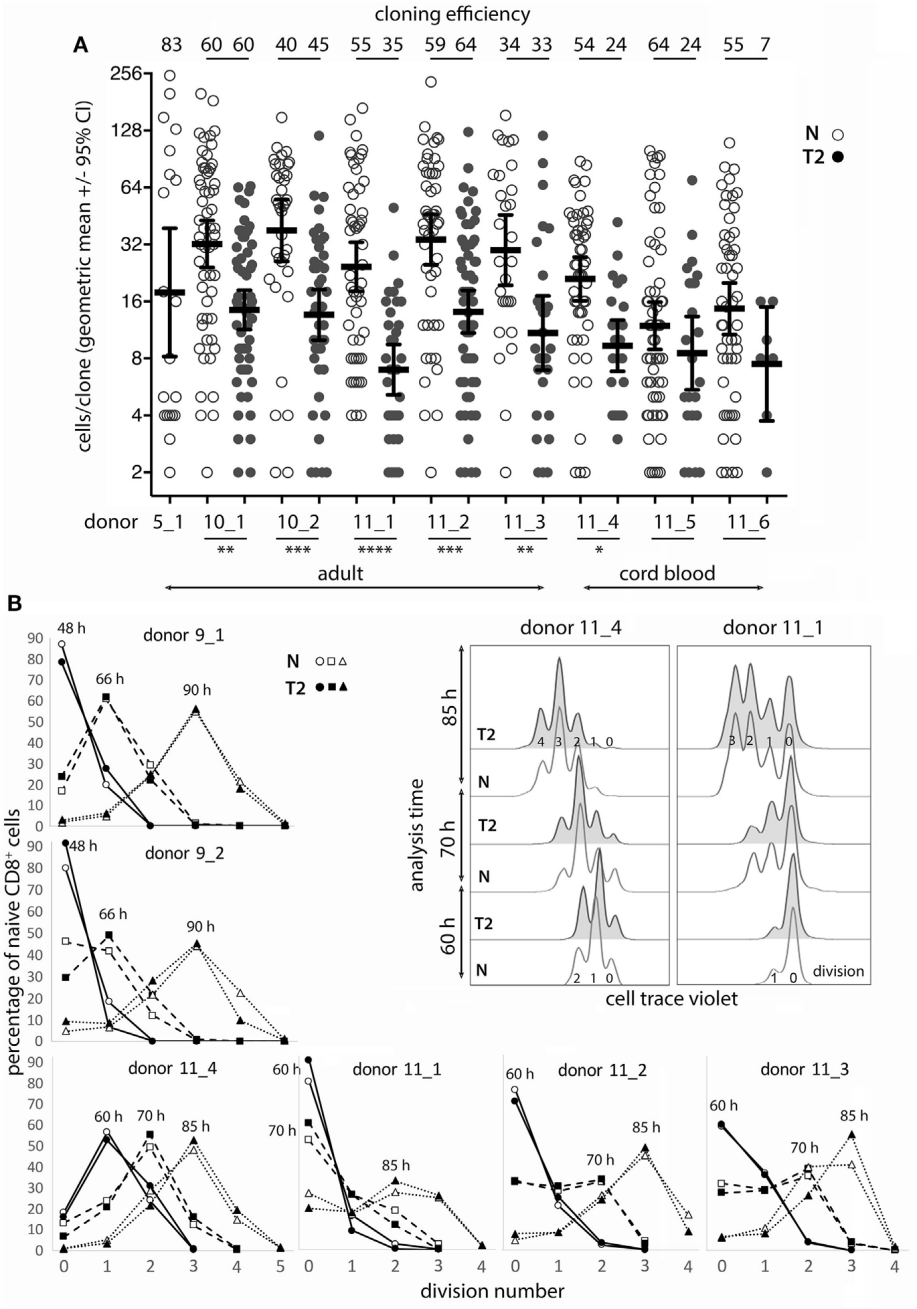
Naïve CD8^+^ T-cell cloning and division in neutral or type-2 (T2) conditions. **(A)** Single naïve CD8^+^ T cells were sorted into mAb-coated 96-well plates and cultured for 6 days in neutral (N) or T2 conditions. Each dot represents a clone and the percentage of single cells that formed a clone (cloning efficiency) is shown above each donor/condition. *p*-Values for within-donor comparisons of clone size in N versus T2 conditions are indicated (****p < 0.0001, ***p < 0.001, **p < 0.01, and *p < 0.05). **(B)** Cells from six donors were cell trace violet (CTV) labeled and cell division was assessed at various times between 48 and 90 h after activation in N and T2 conditions. Overlay histograms show CTV staining (2 of 6 donors shown), and line graphs show percentages of CD8^+^ T cells in each division. Analysis times are indicated above the peak for each line.

Monoclonal Abs to CD11a mimic signaling from cell–cell contact augment T-cell activation (24, 25) and increase murine single CD8^+^ T_N_ cloning efficiency compared with stimulation with anti-CD3 mAb alone (23). We therefore compared human CD8^+^ T_N_ expansion in bulk cultures with mAbs to CD3 (OKT3) and CD11a (MEM83 or HI111) versus commonly used polyclonal stimuli. Cells were activated at varying densities in culture medium containing either IL-2 alone (neutral conditions; N), or IL-2, IL-4, and anti-IFN-γ mAb (T2 conditions; T2). OKT3 with MEM83 induced substantially greater T_N_ expansion from low cell densities than other stimuli, but provided less advantage when starting at higher cell density (Table S1 in Supplementary Material). Accordingly, up to 83% of single T_N_ cells formed clones when stimulated with OKT3 and MEM83 (Figure 1A).

### Effects of Type-2 Conditions on Human CD8^+^ Naïve T-Cell (T_n_) Cloning and Division Following *In Vitro* Activation

As T-cell differentiation is linked to cell division (26), we examined whether T2 conditions affect division by assessing clone formation from single cells and by use of division tracking dye in bulk cultures. The proportion of purified single CD8^+^ T_N_ that formed a clone within 6 days (cloning efficiency) varied between donors, but generally not between neutral and T2 conditions for individual adult donors (Figure 1A). Notably, T_N_ from cord blood had reduced capacity to respond in T2 conditions. The number of cells per clone (clone size) was significantly and substantially smaller in T2 conditions for six of eight donors indicating an effect on division kinetics, cell death, and/or the total number of divisions reached, referred to as division destiny (27). We have not observed any marked effects of T2 conditions on cell viability in our cultures by flow-cytometric analysis of live/dead staining or of propidium iodide versus Annexin V staining (Figure S2 in Supplementary Material). Analysis of cell trace violet (CTV) labeled cells several times between 48 and 90 h after activation indicated little or no effect of T2 conditions on early division kinetics (Figure 1B). Therefore, T2 conditions decrease clonal expansion during primary CD8^+^ T-cell activation, probably by reducing division destiny.

### Effects of Type-2 Conditions on Human CD8^+^ T_N_ Function Following *In Vitro* Activation

**Figure 2:**
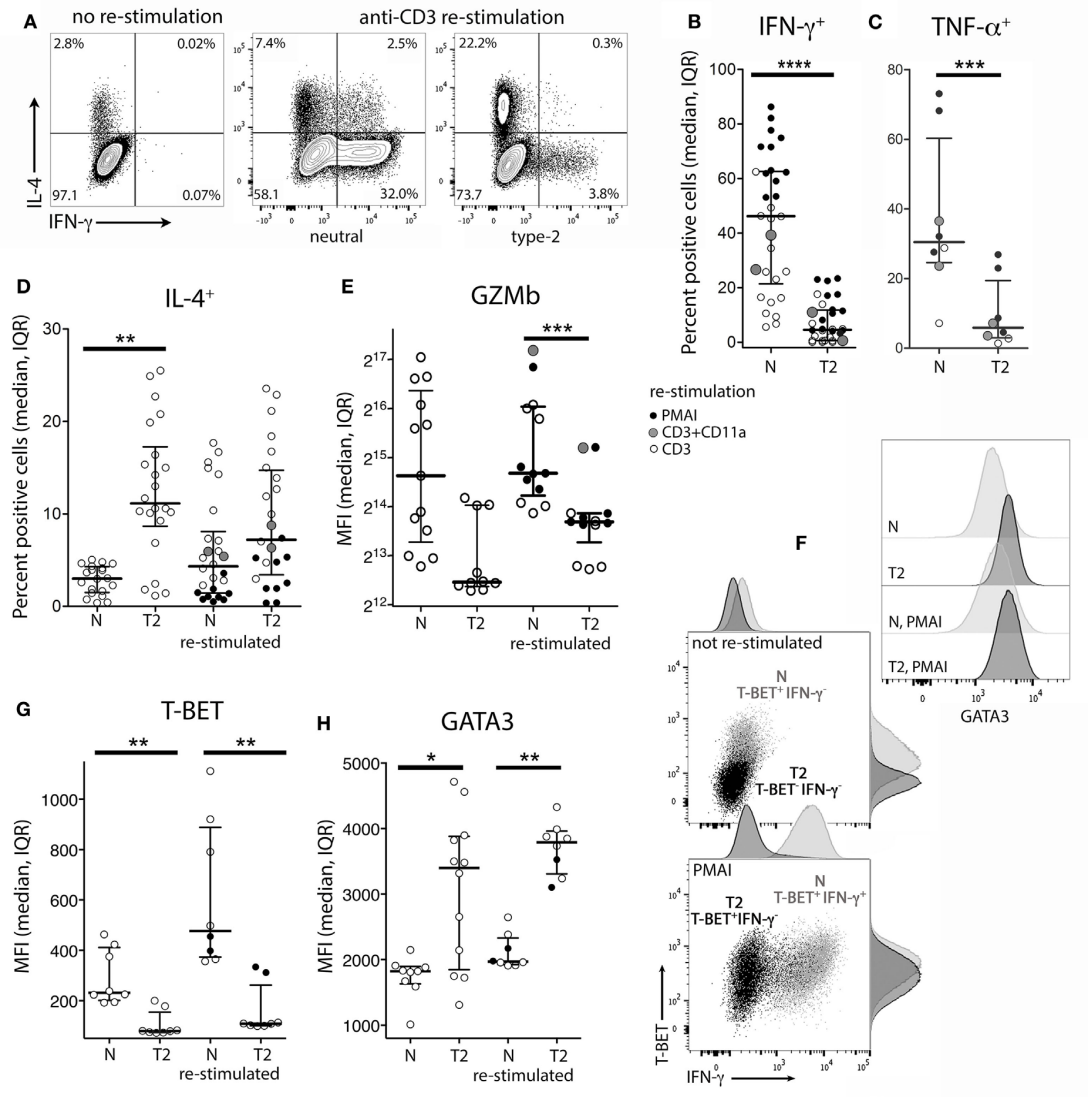
Intracellular cytokine and transcription factor production by CD8^+^ T_N_ stimulated in neutral or type-2 (T2) conditions. CD8^+^ T_N_ were activated in neutral (N) or T2 conditions for 5 days, rested for 3 days, then cultured for a further 5 h with or without restimulation before intracellular or nuclear staining and flow cytometry. **(A)** Representative plots for IFN-γ versus IL-4 staining. **(B–E,G,H)** Each dot represents a sample from up to 13 donors, some of whom were assessed in up to three different experiments. Data are presented as median and interquartile range (IQR). IFN-γ and TNF-α were not detected in non-restimulated cultures (data not shown). *p*-Values for pairwise comparisons of neutral versus T2-primed cells are indicated (****p < 0.0001, ***p < 0.001, **p < 0.01, and *p < 0.05). **(F)** Plots for one of two donors whose cells were costained for IFN-γ, T-BET, and GATA3.

**Figure 3:**
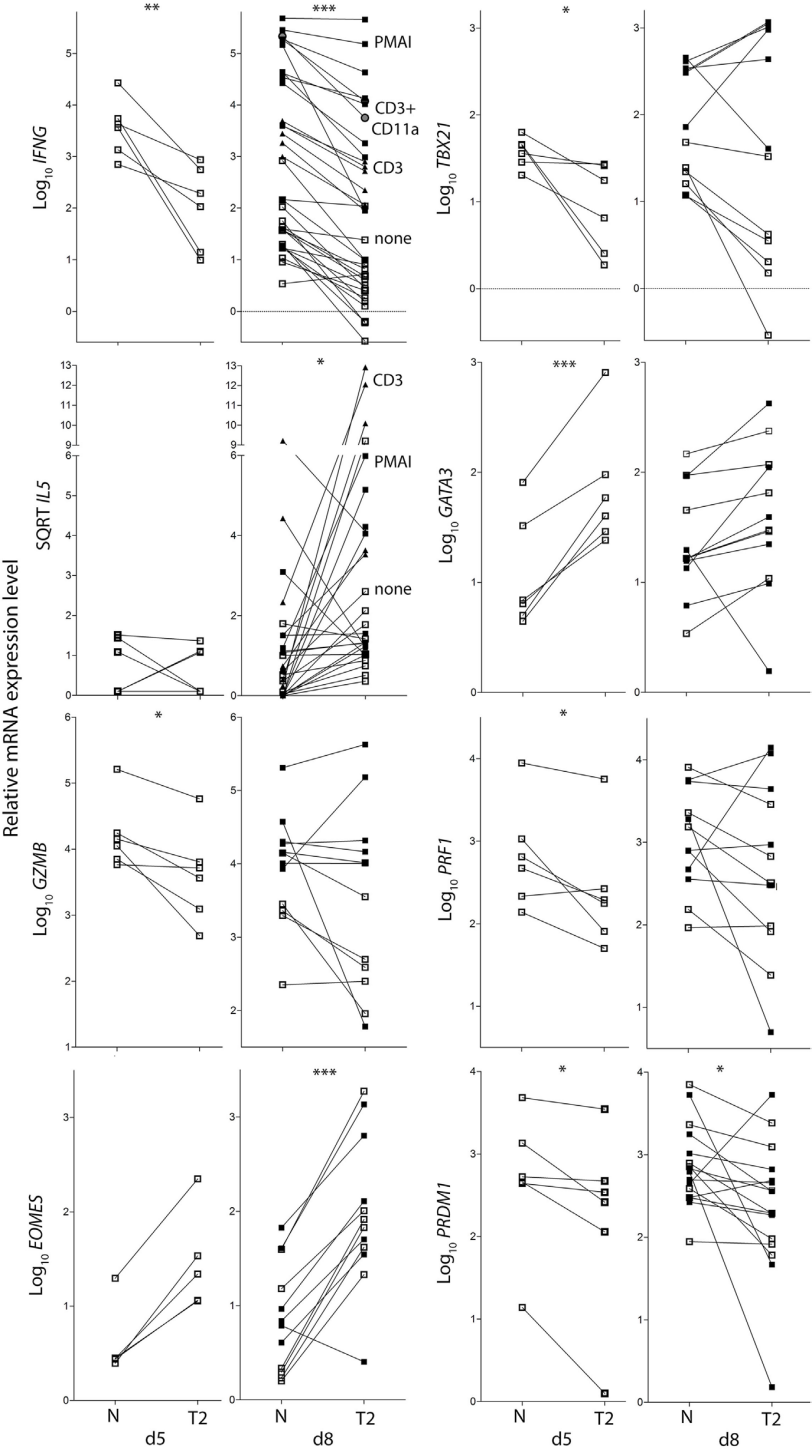
Cytokine and transcription factor mRNA expression in CD8^+^ T_N_ activated in neutral or type-2 (T2) conditions. RNA was extracted from CD8^+^ T_N_ directly after *in vitro* activation in N or T2 conditions for 5 days, or after an additional 3 days of rest with (see below) or without (□) restimulation. Lines join N and T2-cultured cells from the same donor, *n* = 6–12. Cells were restimulated with PMAI (▪), anti-CD3 mAb (▴), or anti-CD3 with anti-CD11a mAb (●). *p*-Values for pairwise comparisons of log_10_ levels in neutral versus T2 conditions are indicated (***p < 0.001, **p < 0.01, and *p < 0.05).

To examine the effects of activation in T2 conditions on T-cell function, flow cytometry and real-time RT-PCR were used to assess expression of cytokines, cytolytic molecules, and associated transcription factors, either after 5 days stimulation, or after a further 3 days of rest followed by 5 h with or without restimulation. CD8^+^ T_N_ activation in T2 conditions resulted in lower production of IFN-γ, TNF-α, Granzyme B (Gzmb), and T-BET protein than in neutral conditions (Figures 2A–G) with consistent effects on corresponding mRNA levels (Figure 3). GATA3 expression was increased and significantly more cells produced IL-4 in T2 conditions, although the percentage of IL-4^+^ cells did not exceed 25.5% (Figure 2D). Perforin (*Prf1)* mRNA levels were also lower in T2 conditions (Figure 3). Regardless of priming conditions, IFN-γ protein and mRNA production were highest when cells were restimulated with PMA and ionomycin (PMAI), followed by anti-CD3 with anti-CD11a mAbs, then anti-CD3 mAb alone (Figure 2B; Figure S3 in Supplementary Material). The reverse was observed for IL-4, consistent with studies showing that higher signal strength favors IFN-γ over IL-4 production (28, 29). Although IFN-γ production by T2 primed then PMAI restimulated cells coincided with T-BET induction, the majority of T-BET positive cells were IFN-γ negative (Figure 2F). In contrast, neutral primed then PMAI restimulated cells that were T-BET^+^ cells were mostly also IFN-γ^+^ (Figure 2F). This suggests that T-BET induction has more effect on IFN-γ expression in neutral than in T2-primed cells.

*IL4* mRNA was barely detected in activated CD8^+^ T cells (data not shown). *IL5* mRNA was more readily detected than IL-4, albeit at low levels compared with type-1 cytokine mRNA, and was higher in T2-primed cells from some donors (Figure 3). Rested T2-primed cells also contained relatively high levels of GATA3 protein and mRNA, with only modest boosting upon restimulation (Figures 2F,H; Figure 3).

The data show that activation of CD8^+^ T_N_ in T2 conditions favors expression of T2 cytokines and GATA3, and lower expression of type-1 cytokines, *Prf1*, Gzmb, and T-BET compared with neutral conditions. IL-4 itself, however, was only weakly expressed at the mRNA and protein levels under T2 conditions, and restimulation with PMAI favored IFN-γ over IL-4 production by both T2 and neutral primed cells.

### Effect of Type-2 Conditions on CD8^+^ T_N_ Transition to Memory/Effector Phenotypes

**Figure 4:**
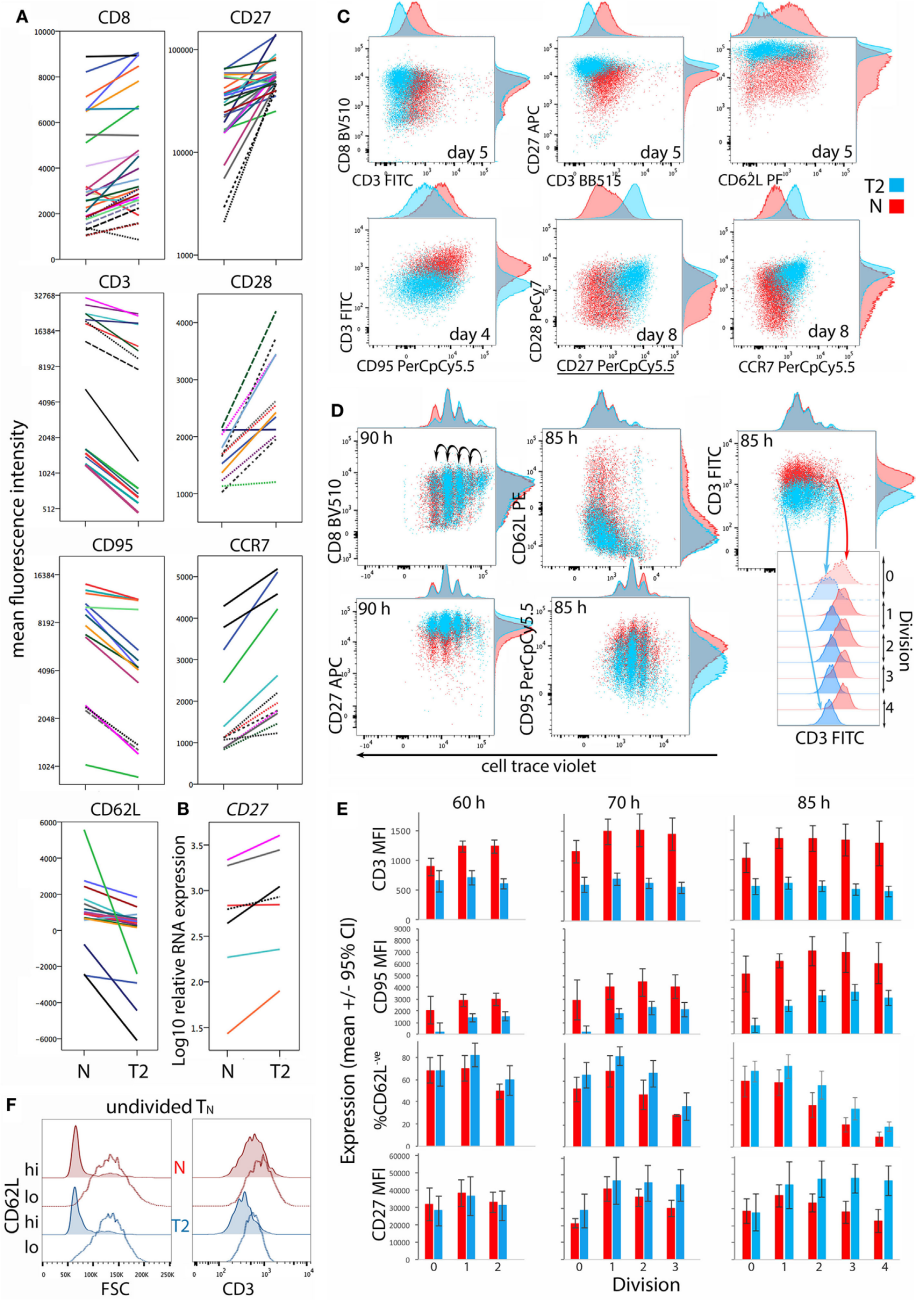
Differentiation phenotype of CD8^+^ T_N_ activated in neutral or type-2 (T2) conditions. CD8^+^ T_N_ were stimulated as described in Figures 2 and 3, and analyzed 5–8 days later **(A–C)**, or were stained with cell trace violet (CTV) and analyzed 60–85 h later **(D–F)**. **(A)** Lines join samples from the same donor and time-point primed in neutral and T2 conditions; dashed lines indicate samples assessed following restimulation. **(B)** *CD27* mRNA expression 5 days after activation, presented as in **(A)**. **(C,D)** Representative overlay plots compare cell surface marker expression for neutral (red) and T2 (blue)-primed cells. **(E)** Surface receptor expression according to division number. Mean and 95% confidence interval for cells from four donors and three time-points are shown. **(F)** Profiles of neutral versus T2-primed undivided CD62L^hi^ and CD62L^−^ cells. Results are shown for one of four donors at 60 h.

CD8^+^ T_N_ differentiation into effector and memory subsets is largely regulated by Blimp1/*Prdm1*, Eomes, and T-BET (30–34). *Prdm1* mRNA levels were lower and *Eomes* levels were higher in T2-primed cells (Figure 3), potentially favoring memory over effector differentiation. We therefore examined whether T2 conditions affect CD8^+^ T_N_ transition to memory and effector phenotypes, which includes progressive loss of CD27, CD45RA, CCR7, CD28 and CD62L (costimulatory or lymph-node homing receptors), and acquisition of effector and pro-apoptotic molecules such as CD95. Progression toward a CD27^lo^ CD28^lo^ CCR7^lo^ CD95^+^ phenotype was arrested in T2 conditions, accompanied by a paradoxical decrease in CD62L expression (Figures 4A,C). *CD27* mRNA was similarly increased in T2 compared with neutral primed T cells (Figure 4B). CTV analysis demonstrated that CD62L was lost early (Figures 4D,E). Approximately 70% of undivided cells were CD62L^lo/−^ (Figure 4E) and undivided CD62L^lo/−^ cells were larger than CD62L^hi^ counterparts (Figure 4F), suggesting they had undergone activation and blast formation, consistent with studies demonstrating that 90% of cells lose CD62L *via* proteolytic cleavage within 4 h of activation (35). The proportion of cells that (re-)expressed CD62L, a signature of central memory precursors (36), then increased as division and time progressed, but this process was impaired in T2 conditions.

CD8α coreceptor expression was examined because downregulation of CD8α mRNA and protein is a hallmark of T2-polarized murine CD8^+^ T cells (9, 37). T2 conditions had no effect on CD8α expression by activated human CD8^+^ T_N_, but CD3 expression was lower in T2 than in neutral conditions (Figures 4A,C). This reflected induction of CD3 upon division in neutral but not T2 conditions (Figures 4D,E). CD3 levels were also higher on undivided CD62L^lo^ FSC^hi^ cells than on undivided CD62L^hi^ FSC^lo^ cells (Figure 4F), indicating that CD3 is induced early after TCR engagement, consistent with reports that cytokine signals influence CD3 levels following TCR stimulation (38). Similarly, T2 conditions prevented induction of CD95 early after activation (Figure 4E). CD27 expression increased during the first few divisions in both neutral and T2 conditions then decreased in T_N_ primed in neutral but not T2 conditions (Figure 4E). Consistent results were obtained with CTV-labeled cells from two additional donors analyzed 66 and 90 h post-activation (Figure S4 in Supplementary Material). Taken together, these phenotypic analyses suggest that CD8^+^ T_N_ differentiation is arrested upon priming in T2 conditions, linked to curtailed division.

### Effects of Type-2 Conditions on CD8^+^ Central Memory T-Cell (T_CM_) Division and Differentiation

The results presented above indicate that CD8^+^ T_N_ division and differentiation commence but are then arrested in T2 conditions. To examine whether CD8^+^ T_CM_ retain sensitivity to the cytokine environment during recall responses, cells with a T_CM_ phenotype (CD45RA^lo^ CD27^hi^) were purified and activated as per T_N_.

**Figure 5:**
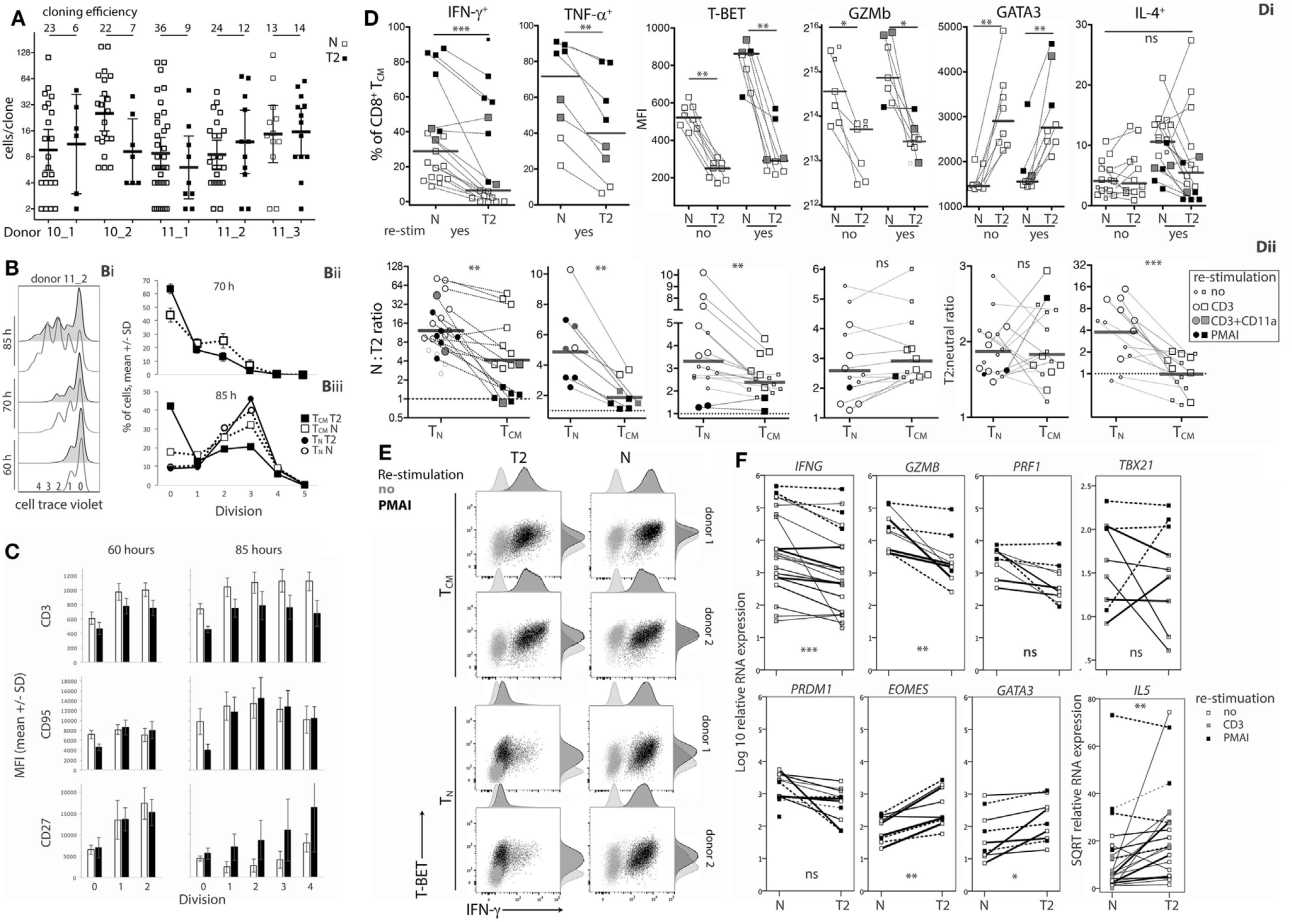
Effects of type-2 (T2) conditions on division and differentiation of CD8^+^ T_CM_ following secondary activation. **(A)** Single T_CM_ cell cloning in neutral versus T2 conditions as in Figure 1A. **(B)** Division analysis as in Figure 1B. Line graphs show mean and SD for T_CM_ from three donors at 70 h (Bii) and for T_CM_ and T_N_ from the same three donors at 85 h (Biii). **(C)** Surface receptor expression according to division number. The mean and SD of results for three donors and two time-points are shown. **(D)** Cytokine and transcription factor levels in T_CM_ from each donor, time and restimulation type; lines join neutral and T2 conditions. Levels in T_CM_ and T_N_ in neutral versus T2 conditions are presented as ratios in Dii. Horizontal bars represent medians. **(E)** Overlay plots of IFN-γ versus T-BET staining of cells with and without PMA and ionomycin (PMAI) restimulation, comparing neutral versus T2 conditions for T_N_ and T_CM_ from two donors. **(F)** mRNA levels for T_CM_ activated in neutral versus T2 conditions, as in Figure 3. *p*-Values for pairwise comparisons of neutral versus T2 are indicated (****p < 0.0001, ***p < 0.001, **p < 0.01, and *p < 0.05).

Cloning efficiencies were substantially lower for CD8^+^ T_CM_ than observed above for T_N_ (Figure 1), and were lower for T_CM_ activated in T2 compared with neutral conditions (Figure 5A). Similarly, the proportion of CTV-labeled T_CM_ that entered division by 70 h and particularly 85 h was lower in T2 than in neutral conditions (Figure 5B), suggesting that *in vivo* priming may render T_CM_ less responsive to T2 conditions. However, T_CM_ clones that formed in T2 conditions contained similar cell numbers to neutral clones (Figure 5A) and, unlike T_N_, T_CM_ upregulated CD3 upon activation and division in T2 conditions. Nevertheless, CD3 mean fluorescence intensities (MFIs) were higher in T_CM_ activated in neutral than in T2 conditions. As with T_N_, T_CM_ modulated CD27 expression in response to T2 conditions (Figure 5C; Figure S5A in Supplementary Material), but largely lost the capacity to modulate CD95 expression (Figure 5C).

Type-2 conditions reduced induction of IFN-γ and TNF-α expression by T_CM_ (Figure 5Di), but to a lesser extent than in T_N_ (Figure 5Dii). On average, 55.2% (11.3–93%) of T2-activated T_CM_ produced IFN-γ when restimulated with PMAI compared with 70.4% (40.2–87.7%, *p* = 0.119) of neutral-activated T_CM_ (Figure 5Di). By comparison, only 12.0% (3.2–23.4%) of T2-primed CD8^+^ T_N_ produced IFN-γ when restimulated with PMAI (Figure 2B). T-BET levels were also higher in T_CM_ compared with T_N_ (Figure S5B in Supplementary Material). Moreover, T-BET induction coincided with IFN-γ production in T2-activated T_CM_ (Figure 5E) suggesting that, like neutral primed T_N_, T_CM_ have undergone priming that renders IFN-γ expression more responsive to T-BET. The effect of T2 conditions on *Ifng* mRNA was markedly weaker for T_CM_ than for T_N_, providing further evidence that T_CM_ have less capacity than T_N_ to regulate *Ifng* transcription. The effects of T2 conditions on Gzmb protein (Figure 5Dii) and mRNA and *Prf1* mRNA (Figure 5F; Figure S5 in Supplementary Material) were equivalent for T_CM_ and T_N_, suggesting that the accessibility of these loci to type-1- and type-2-associated transcription factors may not change during differentiation.

Type-2 conditions induced similar fold increases in GATA3 protein and mRNA, as well as *IL5* and *Eomes* mRNA levels in T_CM_ and T_N_ (Figure 5Dii and Figure 5F; Figure S5B in Supplementary Material). Despite this, T2 conditions did not induce IL-4 production by T_CM_, perhaps reflecting the relative abundance of T-BET in T_CM_ and further indicating that cells that have been primed *in vivo* to become T_CM_ are more committed to a Tc1 pathway than their naïve counterparts.

The data show that CD8^+^ T_CM_ retain some capacity to respond to T2 conditions by expressing higher levels of GATA3 and CD27, and lower levels of type-1 cytokines, *Prf1*, Gzmb, and T-BET compared with T_CM_ reactivated in neutral conditions. However, some of these effects were weaker and were accompanied by lower cloning efficiencies than those observed following activation of CD8^+^ T_N_.

### Effects of Type-2 Conditions on CD8^+^ T_N_ Versus CD4^+^ T_N_

**Figure 6:**
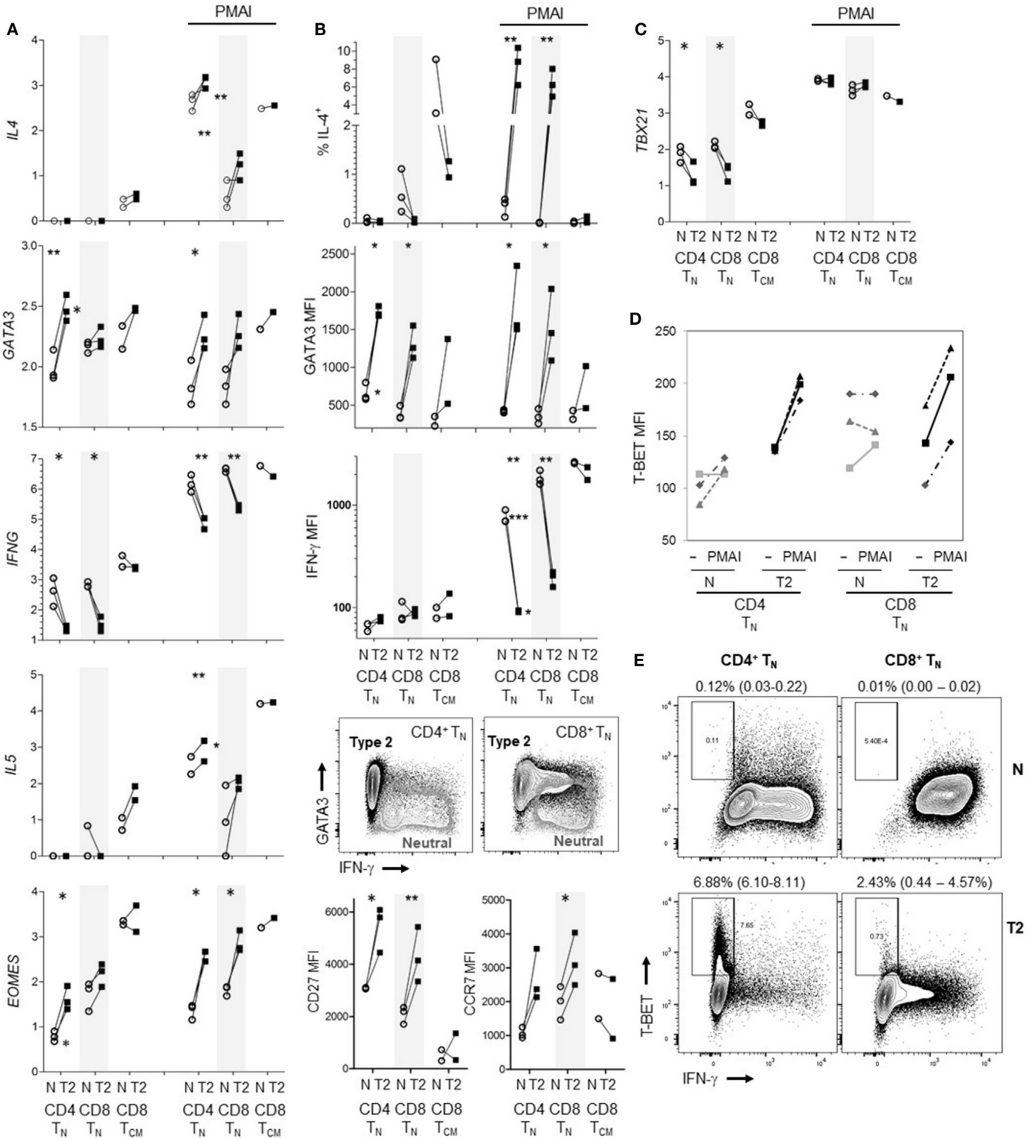
Effects of type-2 (T2) conditions on differentiation of CD4^+^ T_N_ versus CD8^+^ T_N_ and T_CM_ following activation *in vitro*. Sorted T_N_ subsets from three donors and CD8^+^ T_CM_ from two donors were activated in neutral (N) or T2 conditions for 5 days then rested for 3 days. T2 media also contained anti-IL-12 mAb. **(A)** Log_10_ mRNA expression relative to reference gene, with and without PMA and ionomycin (PMAI) restimulation. Lines join results for N and T2-primed subsets from the same donor. *p*-Values are indicated for pairwise comparisons of neutral versus T2 conditions for each subset, as well as for CD4^+^ versus CD8^+^ T_N_ for each priming condition (***p < 0.001, **p < 0.01, and *p < 0.05). **(B)** Intracellular and cell surface protein detection by flow cytometry for each subset, presented as in **(A)**. Representative FACS plots show PMAI restimulated cells from one donor, overlaying profiles for N versus T2-primed cells. **(C)** Log_10_ *TBX21* mRNA expression, presented as in **(A)**. **(D)** Effects of PMAI restimulation on T-BET mean fluorescence intensity (MFI) are compared for each T_N_ cell subset and priming condition. **(E)** Representative FACS plots of IFN-γ versus T-BET staining following PMAI restimulation for each subset and condition for one donor, showing average (range)% T-BET^+^IFN-γ^−^ for three donors.

The results indicate that human CD8^+^ T_N_ have far less capacity to produce type-2 than type-1 cytokines; in particular, T2 cytokine mRNA was barely detectable. However, it has been reported that commitment of human CD4^+^ T_N_ to express Th2 cytokines requires prolonged periods of activation in T2 conditions unlike commitment to produce IFN-γ (4, 39), and studies often use repeated stimulation in T2 conditions to generate human Th2 cells (39–41). We therefore exposed CD8^+^ T_N_ from one donor to two rounds of activation in T2 conditions, for 7 then 4 days with 6 days rest in between. The percentage of cells that were IL-4^+^ reached 22.9% after the initial round of polarization but did not increase after a further round of polarization (Figure S6 in Supplementary Material). We further directly compared the ability of CD8^+^ T_N_ and CD4^+^ T_N_ to produce T2 cytokines when activated in T2 medium augmented with anti-IL-12 mAb. Regardless of priming conditions, CD4^+^ T_N_ from all three donors expressed significantly more *IL4* and *IL5* mRNA than CD8^+^ T_N_ in response to PMAI restimulation (Figure 6A). T2-primed CD4^+^ T_N_ expressed higher levels of *GATA3* mRNA than neutral primed CD4^+^ T_N_ or CD8^+^ T_N_ prior to restimulation (Figure 6A). However, *GATA3* mRNA levels tended to drop after restimulation and did not correspond with *IL4* mRNA levels. For both T_N_ subsets, T2-primed cells expressed more IL-4, GATA3, CD27 and CCR7 protein and *EOMES* mRNA, and less IFN-γ mRNA and protein, at least following PMAI restimulation (Figures 6A,B). IFN-γ MFIs indicated that on average CD4^+^ T_N_ expressed substantially less IFN-γ protein than CD8^+^ T_N_, and if primed in T2 conditions only 1–2% of CD4^+^ T_N_ made detectable IFN-γ compared with 10–18% of CD8^+^ T_N_ (Figure 6B). Accordingly, when T2-primed CD4^+^ T_N_ cells were restimulated with PMAI, *TBX21* mRNA (Figure 6C), and T-BET protein (Figure 6D) were induced, but T-BET^+^ cells did not express IFN-γ (Figure 6E). As noted above, T2 conditions had relatively little effect on cytokine and transcription factor expression by CD8^+^ T_CM_ (Figures 6A–C). Even when primed in T2 conditions, only a minority of CD8^+^ T_N_ and CD4^+^ T_N_ produced IL-4 protein. Thus, the combined results suggest that, while both human T_N_ subsets are pre-disposed to produce type-1 cytokines, the bias toward type-1 cytokine production is stronger amongst CD8^+^ T_N_.

## Discussion

This study set out to determine whether human CD8^+^ T_N_ can differentiate into T2-polarized cells when activated in T2 conditions, reflecting functional plasticity. It was clear that primary activation under T2 conditions reduced differentiation into cells expressing type-1 cytokines, cytolytic molecules, and T-BET and increased the expression of IL-4, IL-5, and GATA3. However, at most 25% of cells could be induced to produce IL-4 and *IL4* mRNA was barely detectable. Moreover, expansion of clones from single cells, differentiation toward an effector phenotype and early CD3 induction were impaired in T2 conditions, reminiscent of effects of T2 conditions on CD4 (42) and CD8α (37) expression levels by murine CD4^+^ and CD8^+^ T_N_, respectively. Studies of mouse T cells demonstrate that this coreceptor tuning attenuates TCR-mediated activation (42, 43). Therefore, the early induction of CD3 may provide a mechanism for CD8^+^ T cells to integrate cytokine signals by fine-tuning TCR signaling and hence division and differentiation. These combined findings support the theory that CD8^+^ T_N_ preferentially respond to and produce type-1 cytokines (5). Accordingly, most T_CM_ appeared to have undergone commitment to a Tc1 phenotype during *in vivo* priming such that far fewer responded upon secondary activation in T2 conditions. Moreover, activation of T_CM_ in T2 conditions did not induce IL-4 and cells continued to produce type-1 cytokines, reflecting heightened T-BET levels and responsiveness.

T-BET and Blimp1/*Prdm1* are required for Tc1 effector generation (30, 31, 33, 34). It is therefore plausible that T2-primed CD8^+^ T cells had reduced capacity to produce IFN-γ, TNF-α, *Prf1*, and Gzmb because they expressed low levels of T-BET and *Prdm1*. T-BET is rapidly induced following TCR and IFN-γ receptor signaling, it binds to the *Ifng* promoter and multiple distal enhancers, bringing them together *via* tethering enzymes that cause the locus to form loops [reviewed in Ref. (44, 45)]. It is likely that similar T-BET-driven *Ifng* locus remodeling occurred when CD8^+^ T_N_ were primed in neutral conditions such that IFN-γ expression correlated with T-BET expression after restimulation with PMAI. In turn, it is likely that there was little *Ifng* locus remodeling *via* T-BET in T2-primed cells because they expressed little T-BET. Consequently, few T2-primed cells expressed IFN-γ after restimulation with PMAI even though T-BET was induced. Similarly, it is likely that T_CM_ have undergone *Ifng* locus remodeling during *in vivo* priming because cells that expressed T-BET after PMAI restimulation also produced IFN-γ, despite secondary activation in T2 conditions.

It has been suggested that epigenetic modifications that permit *Gzmb* and *Prf1* transcription during CD8^+^ T-cell differentiation into effectors are not stably maintained in memory cells whereas modifications in *Ifng* and *Tnfa* loci are maintained (46). The effects of T2 conditions on Gzmb and *Prf1* were equivalent in T_N_ and T_CM_, similarly indicating that transcription-permissive modifications at these loci are not stable. Conversely, *Gzmb* and *Prf1* levels are high in memory compared with naïve cells, corresponding with an open/permissive chromatin state (47). T-BET induces *Gzmb* and *Prf1* expression, and memory cells express high levels of T-BET (47). However, in contrast to *Ifng, Gzmb* and *Prf1* expression require persistent T-BET expression *via* sustained mTOR activation (33, 48); hence, expression by memory cells could be interrupted by mTOR or T-BET downregulation upon exposure to T2 conditions.

GATA3 promotes remodeling of the Th2 locus control region in CD4^+^ T cells (49–51) and was present at higher levels in T2-primed CD8^+^ T cells, so it was somewhat surprising that the latter cells produced little IL-4. However, studies indicate that IL-4 expression is stringently controlled. The probability that recently primed murine Th2 cells will re-produce IL-4 is low, and builds only gradually with successive and prolonged exposure to IL-4 (52, 53), a phenomenon that has been linked to monoallelic *il4* gene expression and to a secondary requirement for NFATc2 binding to the *il4* locus once it has been rendered accessible (54). Our results reveal a similar trend with only low levels of IL-4 mRNA and protein expressed by human CD4^+^ T_N_ under polarizing conditions, and even less expressed by human CD8^+^ T_N_. The *il4* locus is less accessible in mouse CD8^+^ T_N_ than in CD4^+^ T_N_ in association with increased methylation and repressive histone marks (55). Studies in mice also indicate that expression of IL-4 and GATA3 by CD8^+^ Tc2 cells is low compared with CD4^+^ Th2 cells (55–58), and repressor of GATA (ROG) is detected almost exclusively in CD8^+^ T cells (57). Moreover, IL-4 induces only partial demethylation of the Th2 locus control region in Tc2 cells compared with complete demethylation in Th2 cells (59) consistent with indications that CD8^+^ T_N_ are more committed to a type-1 fate than CD4^+^ T_N_ (5, 59). Finally, T-BET redirects GATA3 from Th2 to Th1 genes in CD4^+^ T cells (60) and could have similar actions in CD8^+^ T_N_ which express T-BET. *IL5* mRNA was more readily detected in human CD8^+^ T cells and was induced by T2 conditions, reflecting findings in mice (56). Thus, GATA3 may bind directly to the *IL5* promoter in CD8^+^ T cells as in CD4^+^ T cells (61).

Differentiation toward a CD27^lo^ CD28^lo^ CCR7^lo^ effector phenotype was arrested in T2 conditions, consistent with a study in which IL-4 induced CCR7 expression by CD8^+^ T cells (20). *Eomes* mRNA was induced and *Prdm1* was suppressed in T2 conditions. A similar effect of IL-4 on *Eomes* expression has been reported for mouse CD8^+^ T cells (62). Changes in *Eomes* and *Prdm1* expression are likely to contribute to effects of T2 conditions on phenotype. In particular, *Prdm1*/Blimp1 induces repressive histone modifications in the CD27 loci (63), so *Prdm1* suppression may induce CD27. Similarly, Eomes induction may promote CCR7 expression because suppression of Eomes upon mTOR induction prevents CCR7 expression (33, 64). Although both CD62L and Eomes are associated with T_CM_ development (32), T2 conditions repressed CD62L re-expression, contrary to effects on *Eomes*. It therefore appears that T2-primed cells may be retained in a transitional state, resembling “transitional memory” CD8^+^ T cells that accumulate during chronic HIV (65). It will be important to establish whether a predominance of Th2 cells in the lungs during asthma (66) impairs anti-viral CD8^+^ T-cell differentiation, and contributes to the enhanced severity of respiratory virus infections (66).

Taken together, our findings indicate that while human CD8^+^ T cells can be polarized toward production of T2 cytokines, division and differentiation are impaired, and polarization is reversed upon strong restimulation. This is consistent with the notion that CD8^+^ T cells are predisposed to respond to and produce type-1 cytokines, yielding T_CM_ that respond poorly to reactivation in T2 conditions. Consequently, while exposure to T2 cytokines during activation may impair Tc1 generation, it may not be sufficient for robust Tc2 generation.

## Author Contributions

AF, AK, and KH designed the study. AF and KH conducted the experiments. AF analyzed the data. AF, KH, KK, and AK contributed to interpretation of results. AF drafted the manuscript. All authors critically revised and approved the final version of the manuscript, and agree to be accountable for all aspects of the work.

## Conflict of Interest Statement

The authors declare that the research was conducted in the absence of any commercial or financial relationships that could be construed as a potential conflict of interest.
