# Supplementary material for: Exposure of Human CD8+ T Cells to Type-2 Cytokines Impairs Division and Differentiation and Induces Limited Polarization
Source: Front Immunol. 2018 May 28;9:1141. doi: 10.3389/fimmu.2018.01141 (PMC5985406; doi:10.3389/fimmu.2018.01141)
Supplement: Supplementary file 1 [file table_1.docx]

**Supplementary Table 1. CD8^+^ T_N_ expansion under different activation conditions**

| T cell activation condition^a^ | Fold change in CD8^+^ T-cell number | | | | | | | |
| --- | --- | --- | --- | --- | --- | --- | --- | --- |
|  | Day 5 | | | | Day 9^e^ | | | |
|  | *Starting cell number* | | | | | | | |
|  | 5000 | | 50000 | | 1000 | | 5000 | |
|  | N | T2 | N | T2 | N | T2 | N | T2 |
| OKT3^b^ + MEM83 (CD11a)^b^ | 10.0 | 5 | 11.4 | 11.7 | 121.3 | 28.0 | 123.7 | 39.5 |
| OKT3^b^ + HI111 (CD11a)^b^ | 3.0 | 2 | 4.6 | 5.7 | 34.7 | 20.0 | 43.6 | 26.4 |
| OKT3^b^+ CD28.2^c^ | 1.5 | 0.25 | 6.6 | 5.55 | 2.0 | 10.0 | 26.8 | 9.6 |
| Dynabeads CD3/CD28^d^ | NA | NA | 24.6 | 27.6 | NA | NA | 16.7 | 4.8 |
| PHA | 1.5 | 0 | 1.0 | 0.3 | 0 | 1.0 | 0 | 1.3 |
| PMA and ionomycin (PMAI) | 1.5 | 2 | 0.9 | 0.3 | 0.6 | 18.7 | 0.6 | 6.7 |

a: all cultures included 10 U/ml IL-2

b: mAb clones used at 10 μg/ml to coat plates

c: mAb used soluble at 1 μg/ml

d: Dynabeads CD3/CD28 T Cell Expander (Dynal) used as per manufacturer’s instructions which precludes low cell starting numbers.

e: cells were activated for 7 days then rested for 2 days
