# Supplementary figures and images for: Exposure of Human CD8+ T Cells to Type-2 Cytokines Impairs Division and Differentiation and Induces Limited Polarization
Source: Front Immunol. 2018 May 28;9:1141. doi: 10.3389/fimmu.2018.01141 (PMC5985406; doi:10.3389/fimmu.2018.01141)

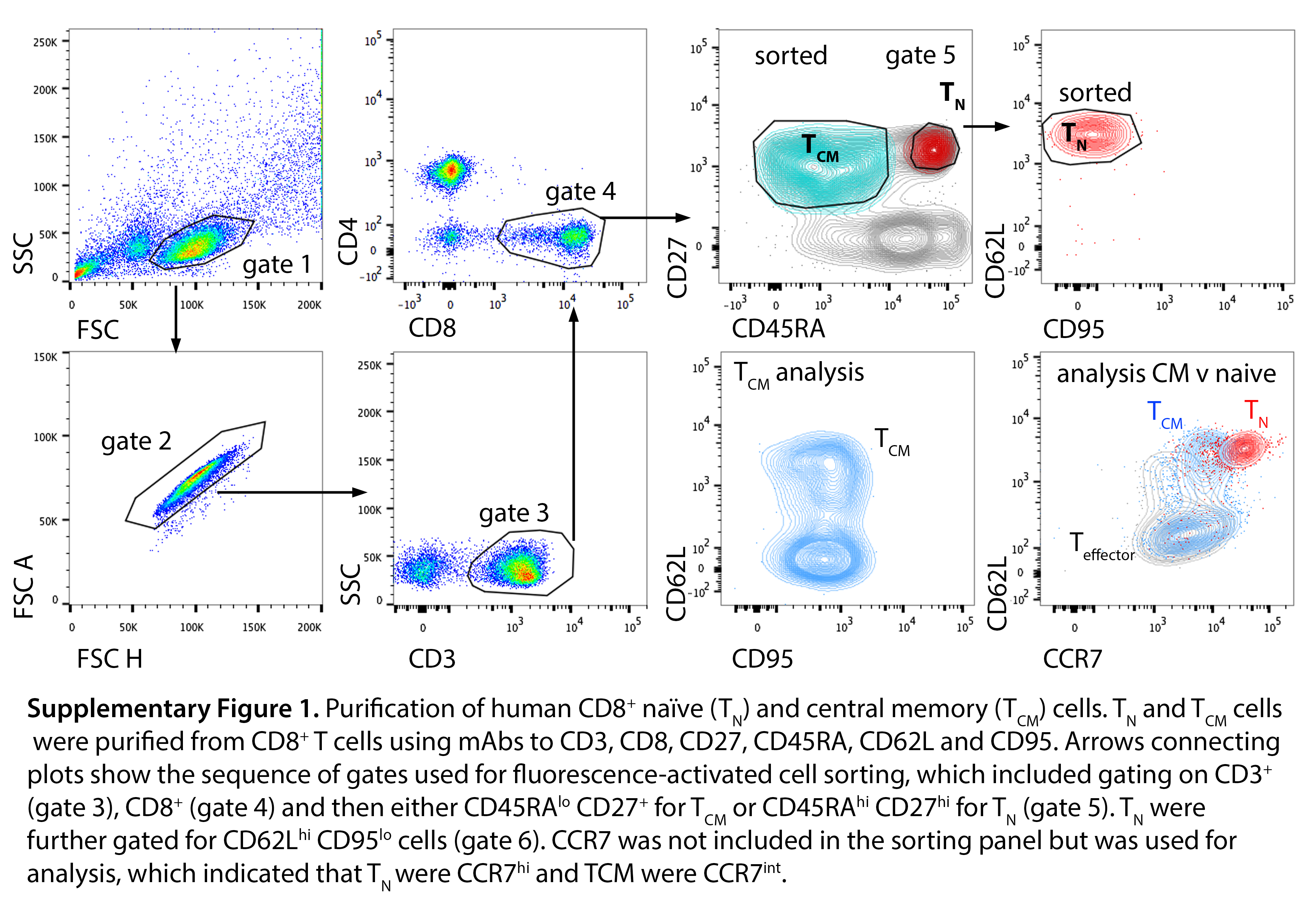

Supplement: Supplementary file 2 [file image_1.tif]

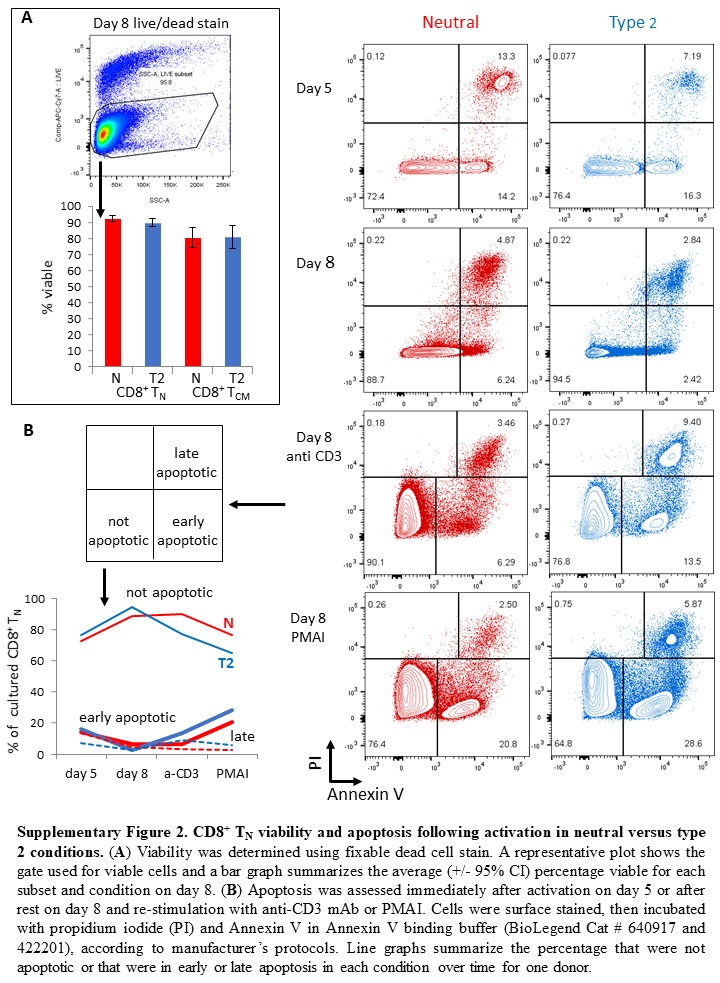

Supplement: Supplementary file 3 [file image_2.jpg]

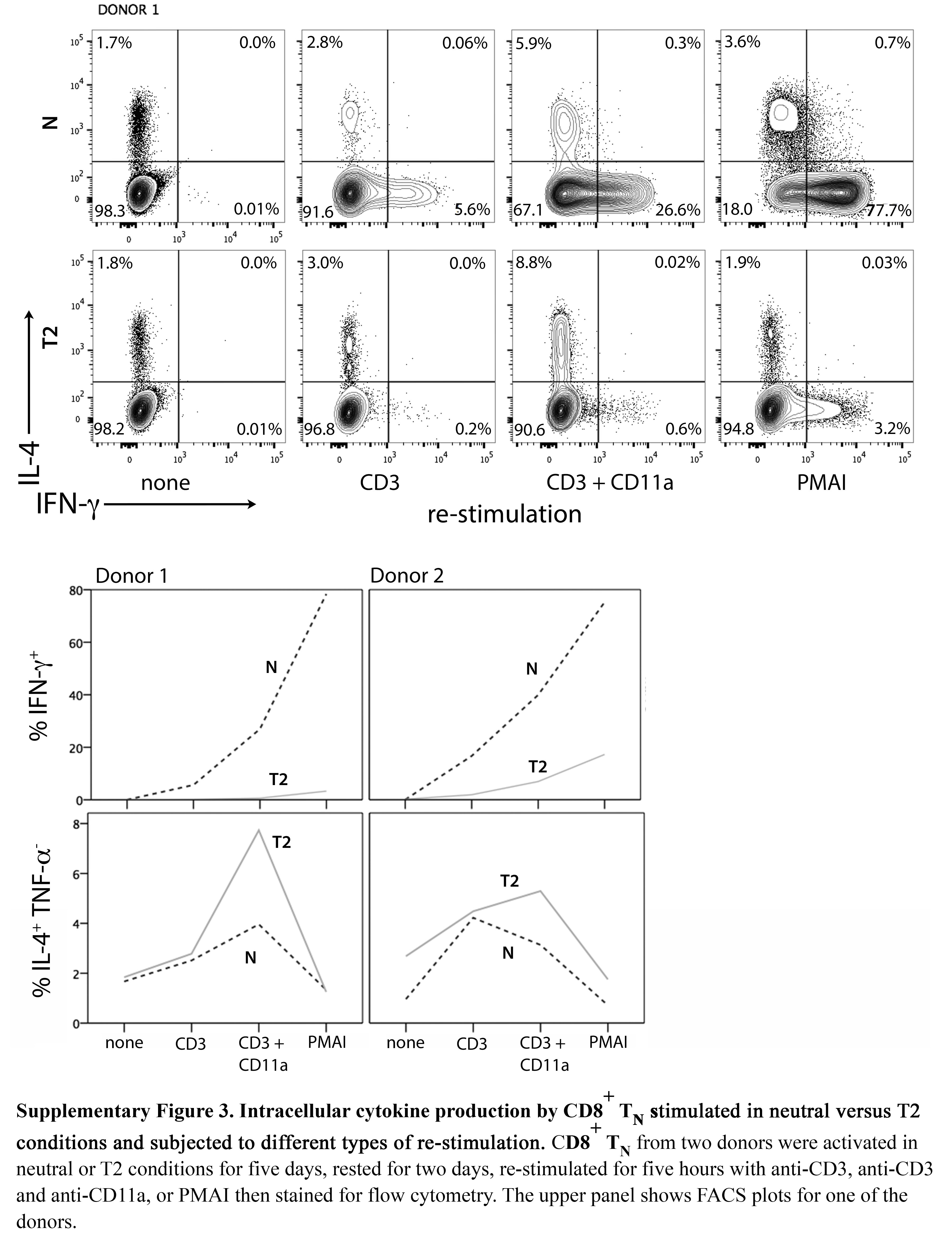

Supplement: Supplementary file 4 [file image_3.jpeg]

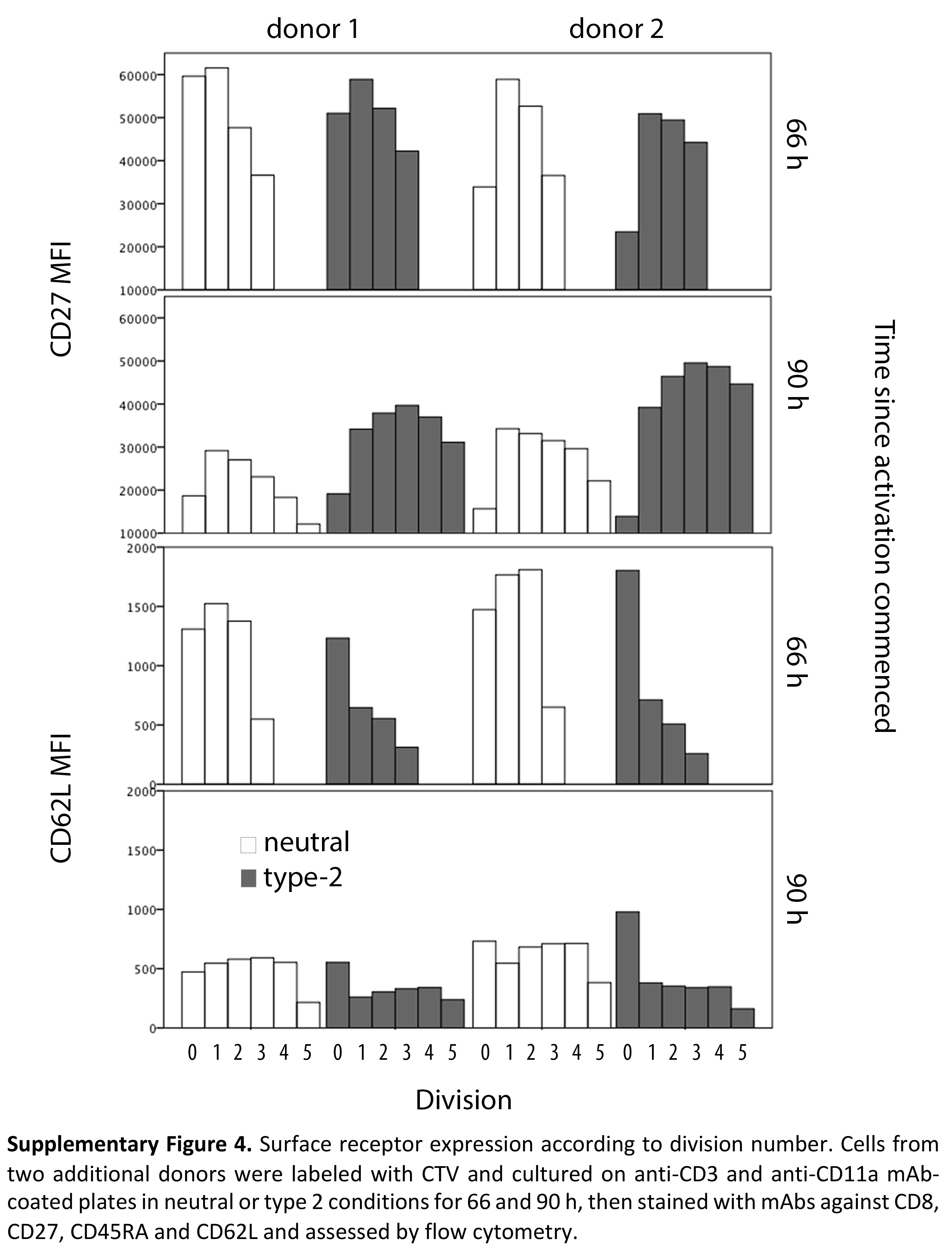

Supplement: Supplementary file 5 [file image_4.jpeg]

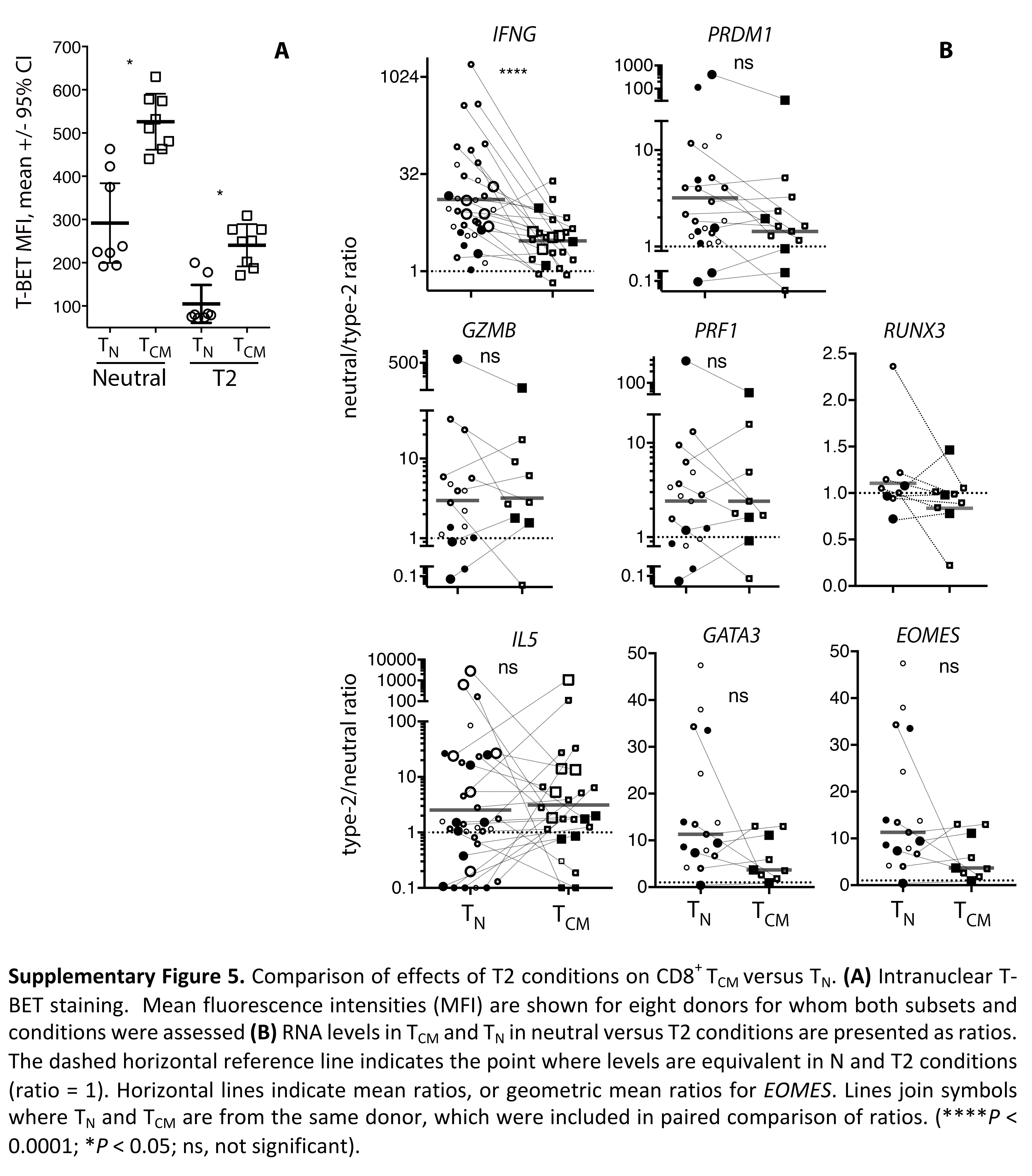

Supplement: Supplementary file 6 [file image_5.jpeg]
